# Cleared to land? A nationwide analysis of emergency care hospital and HEMS infrastructure in Germany

**DOI:** 10.1186/s13049-025-01418-y

**Published:** 2025-06-17

**Authors:** Justus Wolff, Christian Hohenstein, Christian Karagiannidis, Julius Kerkhoff, Hans Morten Lossius, Johannes Strobel, Jakob Ule, Janosch Dahmen

**Affiliations:** 1 Department of Anesthesia, Intensive Care and Emergency Medicine, Military Hospital Berlin, Scharnhorststrasse 13, 10115 Berlin, Germany; 2 Prehospital Emergency Medicine Board, German Society for Emergency Medicine, DGINA e.V., Hohenzollerndamm 152, 14199 Berlin, Germany; 3 Department of Medicine, Philipps-University Marburg, Baldingerstrasse, 35032 Marburg, Germany; 4 Department of Pneumology and Critical Care Medicine, ARDS and ECMO Centre, Cologne-Merheim Hospital, Ostmerheimer Strasse 200, 51109 Cologne, Germany; 5 Emergency Medical Services Working Group, German Society for Emergency Medicine, DGINA e.V., Hohenzollerndamm 152, 14199 Berlin, Germany; 6 Norwegian Air Ambulance Foundation, Postboks 414 Sentrum, 0103 Oslo, Norway; 7 Department of Health, Ethics and Society, Faculty of Health Medicine and Life Science, Care and Public Health Research Institute, Maastricht University, Minderbroedersberg 4-6, 6211 LK Maastricht, Netherlands; 8 Department of Anesthesiology, Intensive Care and Pain Therapy, Saarland University Medical Center, Kirrberger Strasse 100, 66421 Homburg (Saar), Germany; 9 Department of Medicine, Health Faculty, University Witten/Herdecke, Alfred-Herrhausen-Strasse 50, 58455 Witten, Germany

**Keywords:** Emergency Medical Services [MeSH], Air Ambulances [MeSH]; Emergency Service, Hospital [MeSH], Health Services Accessibility [MeSH], Hospital Infrastructure; Helipad; Landing site, Healthcare Centralization

## Abstract

**Background:**

Healthcare systems are increasingly shifting toward specialization and centralization. As a result, distances are growing between emergency patients and suitable emergency hospitals, as well as in between hospitals for interhospital transfers. Helicopter Emergency Medical Services (HEMS) are essential in maintaining equitable access to emergency care, particularly in rural regions. However, the availability and quality of HEMS landing infrastructure at hospitals remains largely unexamined. This study provides the first nationwide integrated mapping and analysis of emergency care hospital and HEMS landing facility distribution.

**Methods:**

We conducted a nationwide cross-sectional analysis of all German hospitals classified under the Emergency Care Level system (ECL I–III). Using data from hospital quality reports, government registries, and satellite imagery, we assessed the availability and type of HEMS landing facilities, categorized as certified helipads or Public Interest Sites (PIS). The study aimed to map and characterize the emergency care hospital and HEMS infrastructure, identify associated hospital and regional factors, and assess spatial access and data completeness through targeted analyses.

**Results:**

Of 1,037 emergency care hospitals, 69.6% have a designated landing facility, with 44.0% of these featuring a certified helipad and 56.0% relying on PIS. A substantial proportion of hospitals (30.4%) lack any HEMS landing facility, especially in urban areas. Certified helipads are more prevalent at higher-tier emergency hospitals (ECL II and III) but no landing facility is available at 18.3% of these facilities, particularly in metropolitan regions. Hospitals in rural areas are more likely to have a HEMS landing facility.

**Conclusions:**

Despite the crucial role of HEMS in emergency medical care, nearly one-third of Germany’s emergency care hospitals lack designated landing facilities, with PIS still outnumbering certified helipads. This reflects structural and regulatory shortcomings that may compromise timely access to specialized care. Enhancing national oversight, modernizing infrastructure, and adopting harmonized European standards are key measures to ensure reliable aeromedical access – and to improve patient outcomes across borders.

**Supplementary Information:**

The online version contains supplementary material available at 10.1186/s13049-025-01418-y.

## Background

Access to medical care plays an essential role in guaranteeing equal living conditions across regions with varying population densities and geographical features. Currently, extensive reforms of the health sector with a trend toward centralization and increasingly different scopes of care is underway in many countries. This creates both a greater necessity for transportation between medical facilities and also greater distances to cover [1–3]. Additionally, recent data indicate that the growing EMS call volume is not primarily driven by low-acuity cases, but rather reflects a more complex demand dynamic across the population [4]. In this context, the role of Helicopter Emergency Medical Services (HEMS) becomes increasingly critical [5]. HEMS helicopters, with their ability to provide critical care transport, are uniquely suited to bridge distances while offering significant time advantages when landing facilities are available. In addition to interhospital transfers (secondary missions), HEMS are progressively vital for those primary missions which require fast response times, highly specialized medical personnel and advanced medical equipment. Following initial treatment, critical patients often require specialized hospitals, which may be situated at a greater distance. In such cases, airborne transport ensures a swift transfer with high-quality medical care enroute, when designated HEMS landing infrastructure is available. Therefore, given the critical and expanding role of HEMS in ensuring timely access to specialized emergency care, particularly amidst increasing centralization and greater transport distances, understanding the current distribution and quality of HEMS landing infrastructure is urgently needed. Pre-pandemic data shows that the use of HEMS has been steadily increasing worldwide [6].

Against this background, this study provides for the first time a comprehensive nationwide data collection and statistical analysis of both the distribution of emergency care hospitals and corresponding HEMS landing facilities. The aim is to present a status quo for emergency care infrastructure in Germany, providing a benchmark across Europe and beyond, as well as developing and sharing possible solutions that could inform similar efforts in other countries.

## Materials and methods

### Study design

This study was designed as a nationwide, cross-sectional observational analysis to provide an integrated assessment of emergency care hospital and HEMS landing facility distribution in Germany.

### Study goals

General aims included the mapping and characterization of Germany’s emergency care hospital network with prevalence and classification of HEMS landing facilities at emergency care hospitals, and an overview on HEMS helicopter fleet structure. The primary endpoint was to conduct a national comparative analysis of emergency care hospitals with their associated HEMS landing facilities, identifying hospital-specific and regional factors that correlate with the presence and type of landing facility. Secondary endpoints focused on analyzing landing facility availability within ECL subgroups, identifying outliers, evaluating data completeness and validity, landing facility spatial logistics, and illustrating accessibility through ground-based ambulance transport time radii surrounding emergency care hospitals.

### Ethical approval

Approval of this study by an ethics committee was not required due to the use of publicly available non-identifiable secondary data. No human participants, patient records, or personal health information were accessed or used. All data processing and reporting were conducted in accordance with applicable European and German data protection laws, including the General Data Protection Regulation (GDPR), and institutional ethical standards.

### EMS System in Germany

In Germany, Emergency Medical Services (EMS) are organized at the level of the 16 federal states and typically run by regional administrative bodies, such as municipalities or counties. This results in a highly decentralized structure with considerable heterogeneity in dispatch organization, operational procedures, and system integration. EMS is predominantly dual-tiered, consisting of paramedic-staffed ambulances and emergency physician rapid response vehicles or HEMS. HEMS missions are generally dispatched via local or regional dispatch centers. However, due to the large number of independent dispatch centers nationwide, the degree of standardization varies considerably [7, 8]. Furthermore, regional variation in HEMS integration stems from an absence of standardized call triage systems and georeferenced dispatch algorithms that identify the fastest EMS resource based on real-time location and urgency [7]. Similar variability applies to hospital destination decisions. While the general principle is to transport patients to the nearest appropriate facility, the absence of a national framework and the influence of administrative boundaries may lead to inconsistent patient allocation — particularly in missions that cross jurisdictional borders.

### Definitions

#### Certified helipads and Public Interest Sites (PIS)

Certified helipads are defined as “small airfields” in accordance with § 6 of the German Air Traffic Act (Luftverkehrsgesetz; LuftVG) [9]. They are subject to clearly defined regulations and requirements pertaining to safety equipment, marking, and location. This results in a high degree of standardization, safety, and availability during daytime (and night-time) hours. Public Interest Sites (PIS) require significantly fewer specifications, in accordance with § 18 subsection 4 and supplement 3 of the German Air Traffic Regulations (Luftverkehrsverordnung; LuftVO) [10]. After installation, PIS landing sites no longer have to be regularly approved or inspected by an official body. While certified helipads are subject to defined obstacle clearance criteria and often support standard arrival and departure routes requiring Performance Class 1 operations, PIS landing sites may be used under less stringent conditions, including Performance Class 2 or 3. This operational flexibility, however, comes at the cost of increased risk: obstacle clearance in the approach and departure paths is neither standardized nor systematically verified, and may change over time without being communicated to HEMS operators or pilots. Thus, the responsibility for ensuring a safe operation rests entirely with the HEMS operator and crew, based on situational judgment and operational risk assessment as defined by EASA SPA.HEMS.105 [11]. Such sites may currently only be utilized as existing landing facilities as part of a transitional arrangement; the establishment of new PIS landing sites is no longer permitted. A special case exists with regard to the use of military landing facilities in accordance with § 30 LuftVG at hospitals of the German Armed Forces and on properties of the German Armed Forces and Federal Police [9]. In this paper, the term “landing facility” is used to collectively refer to all types of HEMS landing facilities.

#### Hospital emergency care level system in Germany

The tiered Emergency Care Level (ECL) System of German hospitals is a structured classification according to their emergency medical treatment capacity and corresponding care mandate [12]. It differentiates between no emergency capacity, basic emergency care (ECL I), extended emergency care (ECL II) and comprehensive emergency care (ECL III).

### Data collection

In the period from April 2023 to December 2023, data on certified helipads in accordance with § 6 LuftVG were collected through parliamentary inquiries in the federal states and through written inquiries to the responsible state supervisory authorities. The list of Public Interest Sites of the Federal Aviation Authority was retrieved as of 22 Nov 2023 [13]. Population figures for Germany and area data for the federal states were taken from official sources with the German Federal Statistical Office and Statista [14, 15]. A list of all German hospitals, their emergency care levels, and hospital related factors were extracted from official quality reports of the hospitals in accordance with Sect. 108 of the German Social Code Book V (Sozialgesetzbuch V; SGB V), from the year 2022. The list of certified helipads at German hospitals was supplemented using the publicly available list of German HEMS bases [16]. All this data was collected in Microsoft Excel (Version 2402, Build 16.0.17328.20670) and structured by individual hospitals. Additionally, satellite imagery were taken from Google Maps (2024).

### Statistical analysis

Statistical analyses were carried out using Microsoft Excel (Version 2402, Build 16.0.17328.20670) and SPSS (Software Version 28.0.1.1). Quantitative characteristics were analyzed using Pearson's correlation coefficient to illustrate the differences between the federal states in terms of population density. The strength of the correlation was classified from strong (|r|> 0.7) to moderate (0.3 <|r|≤ 0.7) to weak (0 <|r|≤ 0.3), while r = 0 represented no correlation. Ordinal-scaled characteristics were analyzed using the Kruskal-Wallis-Test. Comparisons between two groups with quantitative variables were carried out using the t-test for mean values, and for categorical variables using the chi-square test. Multiple binary logistic regression was used to investigate factors influencing the availability of a landing site at a hospital. These factors were hospital-specific and included the emergency care level, number of inpatient cases per year, number of intensive care beds, number of complex neurological treatments per year, geographical location in a federal state that was part of West Germany (old federal states) or East Germany (new federal states) before German reunification, and location in a city with over 100,000 inhabitants (large cities) or over 150,000 inhabitants (major cities). Negative outliers were identified by residual analysis of the expected value with a deviation greater than 10%. An alpha error of < 0.05 was defined. For the statistical analysis, we assumed that a certified helipad has a higher value than a PIS landing site, which has a higher value than no landing facility. Whenever our data sources showed both a certified helipad and a PIS landing site at the same hospital, only the certified helipad was considered in the statistical analysis.

## Results

### General results

In Germany, there are 1675 hospitals, corresponding to 2.04 hospitals per 100,000 inhabitants, varying between the federal states from 1.55 to 2.56 (*n* = 1675, coefficient of variation [CV] 14.34%). Analyzing hospital density per area, the average is 1.26 hospitals per 100 km^2^ (min. 0.17, max. 6.73, CV 145.15%). A total of 1037 hospitals are classified with an Emergency Care Level (ECL I-III), averaging 1.29 emergency care hospitals per 100,000 inhabitants (*n* = 1037, min. 0.84, max. 1.78, CV 20.63%) and 0.79 hospitals per 100 km^2^ (min. 0.12, max. 4.26, CV 142.8%).

Federal states with lower population densities tend to have a higher number of hospitals per 100,000 inhabitants (*r* = −0.576), which is reflected in moderate negative correlations for hospitals with an emergency care level overall (*r* = −0.42), particularly ECL I (*r* = −0.443), and a weak correlation for ECL II (*r* = −0.17). In contrast, the presence of ECL III showed a positive correlation with higher population density (*r* = 0.331). Likewise, hospitals with higher emergency care levels are significantly more common in large cities, while hospitals with lower ECLs predominate in regions with fewer than 100,000 inhabitants (*p* < 0.001). There was also a strong positive correlation between federal states with a higher population density and a higher number of hospitals per square kilometer (*r* = 0.997).

#### HEMS landing facilities

**Fig. 1:**
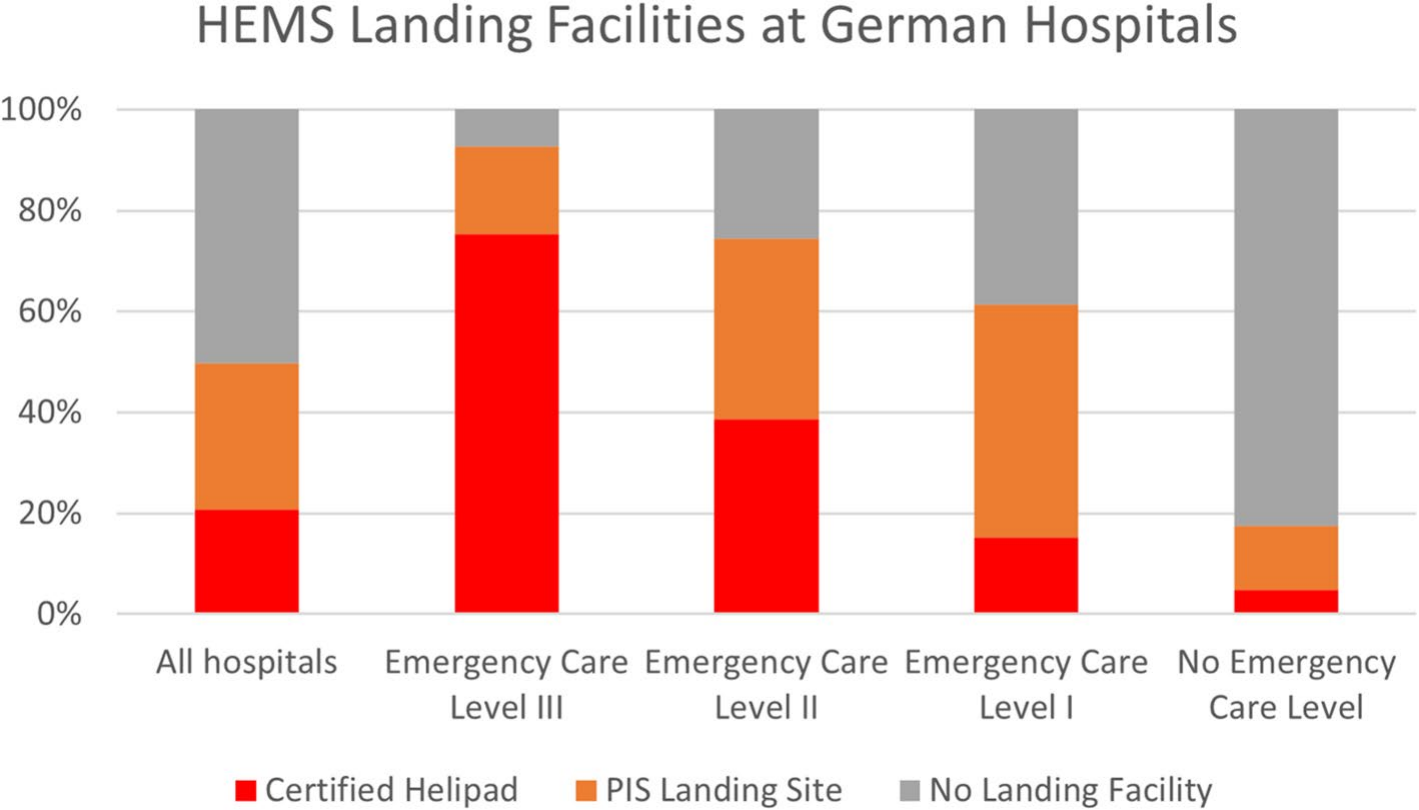
HEMS landing facilities at German hospitals

Out of all 1675 hospitals in Germany, 49.79% (*n* = 834) have a designated HEMS landing facility. Of the 1037 hospitals with an emergency care level, in 69.62% (*n* = 722) of cases a designated landing facility is available. These consist of certified helipads in 44.04% (*n* = 318), while 55.96% (*n* = 404) are PIS landing sites. Simultaneously, 30.38% (*n* = 315) of all emergency care hospitals do not have any landing facility, which is the case in 7.19% (*n* = 12) of ECL III, 25.59% (*n* = 65) of ECL II, and 38.64% (*n* = 238) of ECL I hospitals. Figure 1 shows the distribution of certified helipads and PIS landing sites amongst hospitals with and without emergency care levels. Based on our data, 35 hospitals nationwide showed both a certified helipad and PIS*.* A list of all ECL I–III hospitals with matched landing facility data is provided in Supplement 1. Supplement 2 shows the distribution of landing facilities by federal state and ECL, and Supplement 3 presents an interactive map of Germany with hospitals and landing facilities.

#### HEMS helicopter fleet

In Germany, 84 HEMS helicopters are available for dispatch by rescue coordination centers, comprising rescue helicopters for primary missions, intensive care transport helicopters for secondary missions and dual-use helicopters for both. For 61% (*n* = 51) of these HEMS helicopters, their bases are located at a hospital. Furthermore, there are 5 Search and Rescue (SAR) helicopters belonging to the German Armed Forces and 2 helicopters operated by a private offshore rescue service over the North Sea and Baltic Sea, primarily covering offshore wind farms. In addition to the SAR and offshore rescue helicopters, 16 out of 84 HEMS helicopters in Germany are currently operational on a 24-h basis, most of which are intensive care transport or dual-use helicopters [16]. Four hospitals showed no documented landing facility in our data sources, but a HEMS base on their premises, which were then classified as certified helipads in the statistical analysis.

### Primary endpoints

In a nationwide comparison of emergency care hospitals and availability of landing facilities, the Kruskal-Wallis-Test shows that higher emergency care levels are associated with a higher quality of the landing facility (*p* < 0.001). The Pearson correlation shows that there are fewer landing facilities per hospital in federal states with a higher population density (*r* = −0.69). In large cities, the number of landing facilities per hospital with an emergency care level is lower than in smaller communities with a population of less than 100,000 (*p* < 0.001). The quality of landing facilities across all emergency care hospitals is also significantly lower in large cities (*p* < 0.001). The binary logistic regression analysis confirms that the level of emergency care correlates positively with the existence of a landing facility (OR 1.58, *p* = 0.016) and that in large and major cities there are fewer landing facilities at hospitals (OR 0.30, *p* = 0.003; OR 0.41, *p* = 0.027). The analysis also showed that more landing facilities are found at hospitals in the new (eastern German) federal states compared to the old federal states (OR 2.54, *p* < 0.001). No statistically significant association was observed between the presence of a landing facility at hospitals and the following factors: the number of inpatient somatic cases per hospital, the number of intensive care beds per hospital, and the number of cases involving complex neurological treatment of acute stroke in a stroke unit.

### Secondary endpoints

For hospitals with emergency care levels III, II, and I, an independent negative correlation was observed between population density and the number of landing facilities per hospital (*r* = −0.4; *r* = −0.46; *r* = −0.75). For ECL I and II hospitals, significantly fewer landing facilities per hospital were observed in large cities compared to smaller communities (*p* = 0.001; *p* = 0.001). The analysis by individual emergency care levels shows no correlation in the quality of the landing facility between large cities and other regional authorities. For the subgroup of ECL II and III hospitals, no landing facility is provided at 58 (28.16%) of these hospitals located in large cities and 19 hospitals (16.52%) outside of large cities. For ECL I hospitals, 95 hospitals (70.9%) in large cities do not have a landing facility, compared to 143 (29.67%) outside of large cities. In the identification of negative outliers via residual analysis, the federal states of Schleswig–Holstein, Hesse and North Rhine-Westphalia deviate negatively from the expected prevalence of landing facilities at emergency care hospitals (−19.8%, −16.0%, −14.8%). In the subgroup of hospitals with emergency care level III, negative outliers are Lower Saxony and Hamburg (−12.5% and −11.9%), for emergency level II Saarland, North Rhine-Westphalia, Hesse and Schleswig–Holstein (−48.0%, −19.4%, −18.4%, −13.0%). For ECL I, it is Schleswig–Holstein, Hesse and North Rhine-Westphalia (−37.9%, −18.4%, −10.4%).

**Fig. 2:**
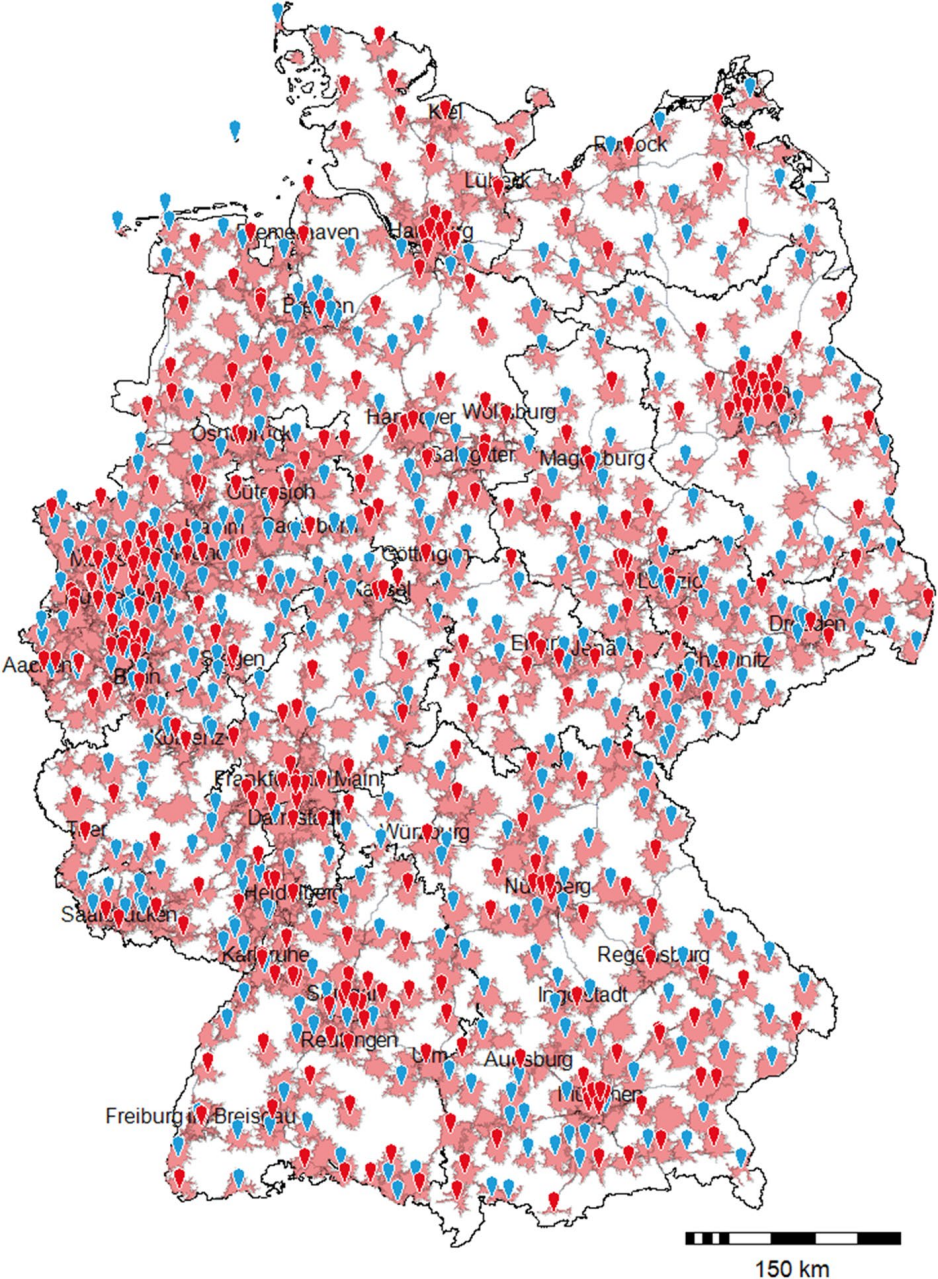
Emergency Care Hospital Availability and Ground-Based Transport Times in Germany. The pins indicate emergency care hospitals with a landing facility (red: ECL II or III, blue: ECL I). The shaded red areas represent the 20-min ground-based transport radii around all emergency care hospitals. Shaded red areas without a pin in the center correspond to emergency care hospitals without a landing facility. White areas represent regions where ground-based transport to the nearest emergency care hospital would take longer than 20 min

Figure 2 illustrates the distribution of emergency care hospitals in Germany, their associated HEMS landing facilities, and areas reachable by ambulance within 20 min under standard road conditions around each emergency care hospital. The transport zones were calculated using routing algorithms based on the underlying road network and predefined movement profiles, which apply average travel speeds assigned to each road segment [17].

As part of the manual review, all hospitals throughout Germany with an emergency care level were looked at with satellite imagery. In the group of emergency care hospitals without a documented landing option (*n* = 315), 42 additional hospitals could be identified that clearly show a landing facility on satellite imagery. Six of these are located on hospital roofs, which makes a certified helipad likely. These additionally identified landing facilities were not included in the statistical analysis. Frequently, a decentralized location of the landing site in relation to hospital grounds and the geotagged emergency room could be observed, with some landing sites more than 1,000 meters away from the hospital building.

## Discussion

This nationwide analysis highlights significant disparities in the availability and quality of HEMS landing infrastructure at German emergency care hospitals. Today, 70% of emergency care hospitals have a designated landing facility – 56% of which are PIS and only 44% certified helipads – while 30% lack any such infrastructure. Following a 2014 political interim solution to reclassify 2,346 helipads as Public Interest Sites (PIS) to circumvent stricter EU safety regulations, certified helipads still remain the exception [18].

### Urban–rural disparities and centralization trends

For a hospital to meet the requirements for ECL II and III, a helicopter landing facility must be provided in accordance with German law. However, this can be bypassed “for reasons that lie outside the hospital's area of responsibility”, for example environmental protection or urban planning regulations [12]. Our analysis for Germany shows that 77 (18.3%) of all ECL II and III hospitals do not have a landing facility. This proves to be more common in large cities, with 28% of ECL II and III hospitals missing a landing facility. It is concerning that even some university hospitals, despite their high level of emergency and specialized care, are missing a landing facility. Collectively, hospitals certified with an emergency care level (ECL I-III) treat approximately 89% of all somatic inpatients in Germany [19]. These findings underscore differences in emergency infrastructure planning, particularly in metropolitan regions where space constraints and regulatory hurdles may impede the establishment of landing facilities. With regard to necessary inbound primary and secondary HEMS flights out of more rural areas, it is worrying if high-care hospitals may only be accessible by ground transport, potentially delaying access to specialized care.

On the other hand, focusing solely on percentages may obscure a broader structural cause: the high absolute number of hospitals in many German cities. From an international perspective, Germany has a very high hospital density of 37.3 hospitals per 1 million inhabitants, shaping both ground-based and air rescue transport needs. In Europe, only France (45.6) and Finland (44.8) have a higher density per 1 million inhabitants, primarily due to sparsely populated areas [20]. Across Europe, hospital density is steadily decreasing, a trend that is particularly pronounced in Germany due to a growing shortage of healthcare resources [20]. While this study did not evaluate the adequacy of hospital distribution in Germany, current short interhospital transport distances likely reflect a historically dense hospital landscape rather than optimized system design.

In a future, more consolidated system, equipping all ECL hospitals with a landing facility would be advisable – particularly for rural hospitals and high-volume urban ECL II/III centers, while smaller ECL I hospitals in major cities may be less dependent on one. Standardization of and proactive investment in HEMS landing infrastructure, especially at smaller rural hospitals, are essential to maintain timely access to advanced care via outbound HEMS flights and enable capacity-relieving inbound transfers from higher-level hospitals [21]. However, a European comparison shows that there is no uniform standard with regard to landing facility regulations, although the European Union Aviation Safety Agency (EASA) is endeavouring to promote uniformity through the Easy Access Rules for Air Operations [11]. While our data did not include details on the presence of auxiliary or diversion landing sites, we acknowledge that such infrastructure may be critical for high-volume hospitals, especially ECL III facilities. The availability of a secondary landing option can help mitigate risks associated with simultaneous helicopter approaches and may prevent mission delays or diversions. Maintaining landing sites at decommissioned peripheral hospitals could provide strategic rendezvous points for HEMS and ground services, especially in rural areas, though sustainable funding models for ongoing operation remain unclear. In the context of national and alliance defence, a resilient network of certified HEMS landing facilities contributes greatly to strategic readiness of critical infrastructure beyond peacetime emergency care.

To ensure that air rescue helicopters – a limited and costly resource – are dispatched only to patients who truly require them, accurate, evidence-based, and standardized emergency call-taking systems are essential [8]. This need becomes increasingly urgent in light of rising emergency call volumes, to support effective and resource-conscious EMS deployment [4]. Currently, the availability of HEMS resources in Germany is still limited in many regions to daylight hours and VFR (Visual Flight Rules) conditions. However, extended HEMS operational hours — particularly during nighttime or off-peak hours — could significantly improve access to time-critical care in rural or underserved areas.

### Persistence of PIS and planning failures

The prolonged reliance on PIS highlights the lack of a strategic modernization plan. Certified helipads offer greater safety and operational reliability but remain underused in favor of the less regulated PIS model, raising concerns about long-term suitability in a changing healthcare system. According to the German Federal Aviation Office, there were almost 30,000 take-offs and landings on PIS across Germany in 2020 [22]. Our data review revealed significant inconsistencies in the official PIS registry, including outdated entries, incorrect coordinates, and listings at closed hospitals or non-functional sites such as parking lots. Following ground-based patient transport, certified helipads enable more flexible retrieval of HEMS physicians after patient handover at the receiving hospital, improving operational continuity. Moreover, many landing facilities were found to be located far from emergency departments, requiring intermediate ambulance transfers that may diminish the time advantage of HEMS and can increase patient risk [23, 24]. Although not quantified in this study, these factors warrant consideration in future infrastructure planning and hospital certification.

### Governance, data quality, and strategic investment

Recent data from Germany demonstrate significant regional differences in the EMS sector, including variations in practice and system-related costs, highlighting the fragmented nature of emergency medical care [7, 25]. The absence of a centralized, current HEMS landing facility registry hampers operations and reflects poor governance – an issue that could be addressed by mandating regular state-level reporting to maintain a national database. The specifications of all HEMS landing facilities could be included in the Aeronautical Information Publication (AIP; Luftfahrthandbuch), for use by HEMS flight personnel.

The observed disparities between old and new federal states, with the latter showing a higher availability of HEMS landing facilities, can be attributed to significant investments made in the 1990s to modernize healthcare infrastructure in the new federal states following German reunification. This reflects the long-term impact of targeted regional development. On a positive note, the newly established Hospital Transformation Fund allocates 50 billion euros over ten years to modernize Germany’s hospital system, including funding for HEMS landing facilities [26]. To cover the operating costs of HEMS landing facilities at hospitals, one possible long-term option would be to implement a flat landing fee. This could be classified as an operating expense for HEMS providers and could therefore be reimbursed by health insurance funds, however, possibly leading to higher insurance premiums and administrative effort.

### International models and technological readiness

The sovereignty of German federal states in EMS policy making effectively leads to a ping-pong of responsibility and prevents comprehensive organization and planning opportunities for HEMS. Other European nations employ a centralized, nationally funded HEMS system with standardized infrastructure [7]. Taking Norway as an example, there is a 47-year history of government-supported helicopter emergency medical services, and in 1988, it became the first country in the world to fully integrate HEMS into its national healthcare system. Today, Norway operates 21 helicopters from 20 bases. A key feature of the system is its standardization: All helicopters – civilian and military – have standardized requirements for staff and medical technology [27]. HEMS plays a growing role due to the centralization of services and a reduction in local hospitals [28, 29]. While landing facility placement at smaller hospitals depends largely on local factors, all high-level (university) hospitals are equipped with certified helipads. Germany’s reliance on PIS landing sites and a decentralized (H)EMS governance model contrasts sharply with Norway’s integrated approach. Adapting elements of this system – such as establishing unified operational standards, funding mechanisms, and centralized governance – could offer a strategic path forward.

From a European perspective, the lack of harmonized HEMS standards contributes to cross-border disparities, particularly in implementing Instrument Flight Rules (IFR) infrastructure — crucial for safe operations in poor weather or at night. Low-Level IFR networks and Point-in-Space (PinS) approaches allow helicopters to navigate to GPS-defined points near hospitals and transition safely to visual landings, even without IFR-certified helipads [30]. However, adoption is limited by infrastructure deficits and regulatory fragmentation. A list with challenges and opportunities relating to HEMS infrastructure matched with policy solution proposals is available in Supplement 4.

As healthcare systems across Europe increasingly centralize and specialize, these advances should be matched by equivalent investments in air transport infrastructure to maintain equitable access to emergency care. Collaborative efforts between policymakers, healthcare providers, and the aviation sector are essential. Future research should incorporate population coverage analyses, as this could provide more precise insights into regional accessibility, and explore the clinical impact of landing facility availability to align aeromedical infrastructure with evolving emergency care delivery models.

### Limitations

The data collected as part of the analysis is based on information provided by the respective state supervisory authorities, but the quality has proven to be limited in some cases. Moreover, this study represents a single moment in the past, within the ongoing transformation of the hospital landscape in Germany. It should be noted that at the time of publication, some hospitals may have been newly established, already been closed or assigned a different emergency care level. A further source of imprecision arises from assigning hospitals to administrative boundaries rather than functional geographic areas, potentially distorting results, especially for facilities on urban peripheries serving rural populations. Despite the implementation of a logistic regression analysis, other influencing factors, such as infrastructural connections or the availability of landing facilities in the surrounding area of a hospital, could not be fully integrated into the models. Furthermore, the presence of selective outliers in the data quality, such as incorrect entries or outdated information on PIS landing sites, may have distorted the results. Hospitals situated at or on airport premises, practically equivalent to a certified helipad, were not accounted for separately.

## Conclusions

This study provides a first nationwide, integrated mapping and analysis of emergency care hospital and HEMS landing facility distribution. We found that nearly 30% of all emergency care hospitals in Germany lack any landing facility, and that Public Interest Sites (PIS) still predominate over certified helipads, falling short of modern safety and operational standards. These findings highlight critical infrastructure gaps that could impair timely access to emergency care. Immediate implications include the need for targeted infrastructure investments, improved regulatory coordination, and integration of landing site data into nationwide registries, to ensure operational readiness, interoperability, and patient safety in both primary and interhospital aeromedical missions.

## Supplementary Information

Supplementary Material 1.

Supplementary Material 2.

Supplementary Material 3.

Supplementary Material 4.

## Data Availability

Data is provided within the manuscript or supplementary information files.

## Abbreviations

ARDS: Acute Respiratory Distress Syndrome
AIP: Aeronautical Information Publication
CV: Coefficient of Variation
ECMO: Extracorporeal Membrane Oxygenation
ECL: Emergency Care Level
EMS: Emergency Medical Services
EU: European Union
G-BA: Gemeinsamer Bundesausschuss, Federal Joint Committee
HEMS: Helicopter Emergency Medical Services
IFR: Instrument Flight Rules
ICU: Intensive Care Unit
LuftVG: Luftverkehrsgesetz, German Air Traffic Act
LuftVO: Luftverkehrs-Ordnung, German Air Traffic Regulations
OR: Odds Ratio
PinS: Point-in-Space
PIS: Public Interest Site
SAR: Search and Rescue
SGB V: Sozialgesetzbuch V, Social Code Book V
SPSS: Statistical Package for the Social Sciences
VFR: Visual Flight Rules
